# The telephone nursing dialogue process: an integrative review

**DOI:** 10.1186/s12912-023-01509-0

**Published:** 2023-09-28

**Authors:** Silje Rysst Gustafsson, Anna Carin Wahlberg

**Affiliations:** 1 Division of nursing and medical technology, Department of Health, Learning and Technology, Luleå University of Technology, Luleå, SE-971 87 Sweden; 2 Division of Nursing, Department of Neurobiology, Care sciences and Society, Karolinska Institutet, Stockholm, SE- 171 77 Sweden

**Keywords:** Telephone nursing, Telephone advice nursing, Dialogue process, Telephone triage

## Abstract

**Background:**

Telephone nursing involves triage, advice, and care management provided by a nurse over the telephone. The telephone nursing dialogue process has been used clinically in telephone nursing in Sweden for several years to structure the communication and ensure a safe assessment and advice. Studies are needed to determine whether there is sufficient scientific evidence to support the method.

**Aim:**

To describe the scientific basis of the phases of the telephone nursing dialogue process.

**Design:**

This was an integrative review.

**Methods:**

The literature searches were performed in August 2023, in the PubMed, CINAHL, Cochrane Database of Systematic Reviews and SwePUB databases. Sixty-two articles were included. Data was sorted deductively according to the five phases of the telephone nursing dialogue process and categorized inductively to form subcategories describing the content of each phase.

**Result:**

All five phases in the telephone nursing dialogue process were supported by a range of articles (n = 32–50): Opening (n = 32), Listening (n = 45), Analysing (n = 50), Motivating (n = 48), and Ending (n = 35). During the opening of the call, the nurse presents herself, welcomes the caller and establishes a caring relationship. In the listening phase, the nurse invites the caller to tell their story, listens actively and confirms understanding. During the analyzing phase, the nurse gathers, assesses, and verifies information. In the motivating phase, the nurse reaches a final assessment, informs the caller, gives advice and creates a mutual agreement and understanding while supporting the caller. Ultimately, the nurse ends the call after checking for mutual agreement and understanding, giving safety-net advice, deciding on whether to keep monitoring the caller and rounding off the call.

**Conclusion:**

The phases of the telephone nursing dialogue process as described in the scientific literature are well aligned with the theoretical descriptions of the telephone nursing dialogue process.

## Introduction

Telephone nursing (TN) is the provision of nursing care over the telephone [1] and involves telephone triage, telephone advice, and care management provided by a nurse [2] Telephone triage is defined as “a complex process of identifying a patient’s problem, estimating the level of urgency, and rendering advice over the phone, while ensuring the safe, timely, and appropriate management of patient symptoms” [3]. Care management implies that the nurse assists the caller and/or their support systems in managing medical conditions [4]. The contact with the TN service usually results in health information about bodily functions, symptoms, risks and/or medications [5–7]as well as advice to visit an emergency department, to see a general practitioner or other healthcare provider, or to perform self-care [2].

## Background

Many countries have introduced national TN services as a first portal to healthcare to optimize the use of health-care resources. Reports on the effectiveness of TN in cost-reduction reveal that TN is comparable to traditional care [8], has the potential to reduce the number of immediate visits to doctors and does not increase visits to emergency departments [9]. Data from Sweden suggest that TN contributes to a shift in healthcare visits from secondary to primary care, indicating that TN increases system efficiency [10]. TN results in costs similar to those of traditional care [11], the safety of TN is equal to that of traditional care [8], and has patient satisfaction comparable to, or higher than, satisfaction with traditional care [8]. Swedish Healthcare Direct (SHD) is the national TN service in Sweden. It is available around the clock, is free of charge and receives approximately 11,000 calls per day [12]. Nurses working at the SHD are often experienced specialist nurses and have received training in using the telephone nursing dialogue process.

**Fig. 1:**
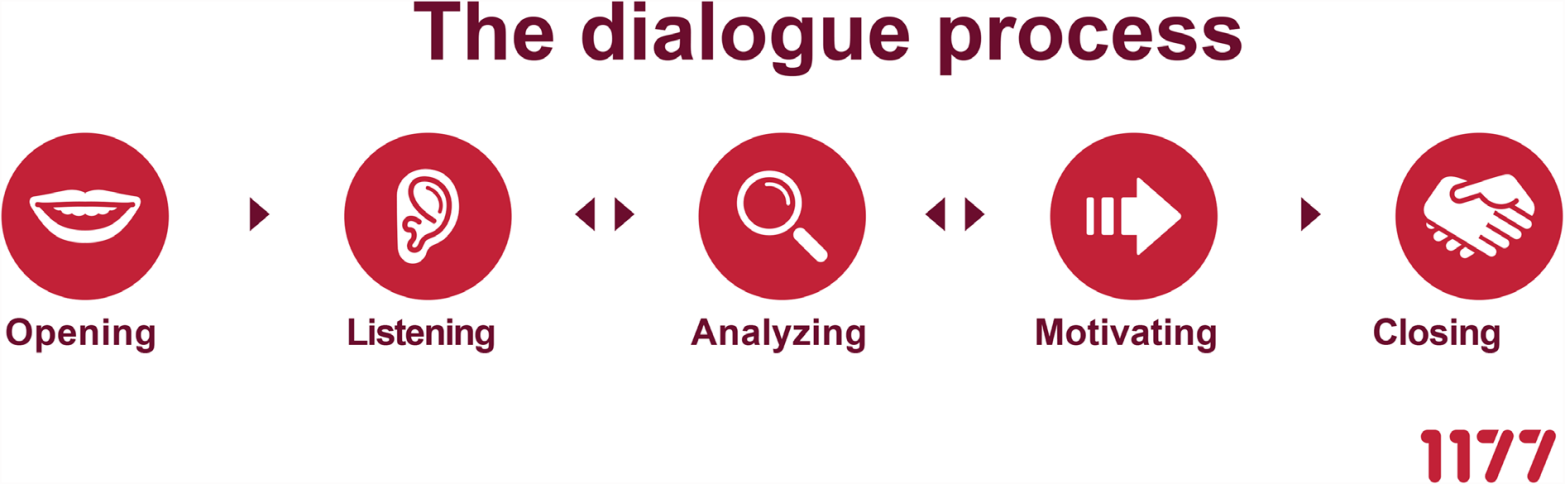
The telephone nursing dialogue process

The nurses working at SHD follow a specific communication process called the telephone nursing dialogue process when handling a call [13]. This method provides structure to the call and supports the systematic exploration of the situation to obtain a relevant picture and sufficient information as a basis for a safe assessment and advice [14]. The telephone nursing dialogue process, as described by Runius [14, 15], is a process consisting of five phases: *Opening, Listening, Analyzing, Motivating* and *Ending* (Fig. 1). The *Opening* phase creates the foundation for good communication and a trusting relationship. The nurse opens the conversation with a welcoming voice that conveys empathy and interest and demonstrates that they have time to listen. In the *Listening* phase, the nurse focuses on the caller and listens actively to the caller’s concerns. The nurse confirms that the caller is being heard with supporting sounds like “*Aha*”, “*OK*?”, “*I see*” without interrupting the caller. When the caller has finished, the nurse makes a brief summary of what the caller has said to check that the caller’s concern has been correctly understood. The nurse then transitions to the *Analyzing* phase to explore the caller’s concern with an open mind, asking both open and closed questions. Short summaries are continuously made to check understanding. Medical history and medications are mapped, and the nurse strives to understand the callers concern from a holistic perspective by also exploring the caller’s context, thoughts and fears. At the end of the Analyzing phase, the nurse makes an overall summary. The call then enters the *Motivating* phase, where the final assessment is presented, explained and justified so that the call output so that the caller will feel motivated. The nurse explores whether the caller understands the assessment and advice, and that consensus prevails. The last phase of the call is the *Ending* phase, where safety-net advice is given before the call is ended. This implies giving information about what symptoms the caller should look out for that require a new assessment before ending the call [14, 15].

The telephone nursing dialogue process was created during the 1990s and builds on the conversational techniques of motivational interviewing [14]. These techniques involve asking open-ended questions and using affirmations, reflective listening and summaries. Asking open-ended questions involves asking questions that are not easily answered with a yes or no, and that invite the caller to elaborate about the concern. Affirmations are statements that confirm to the caller that they are being listened to and that recognize the caller’s emotions or efforts, such as “*That sounds painful*”. Reflective listening involves expressing empathy and understanding by careful listening and reflective responses (i.e. support signals) [16]. Support signals convey presence, involvement and interest [14]. Summaries are recaps of information that are used to communicate interest, understanding and attention [16]. The telephone nursing dialogue process incorporates all of these techniques, and active listening has a central role in the communication [14].

Communication failure is the most common cause of malpractice claims and imposes a considerable threat to patient safety [17, 18]. A lack of structure in the conversation has been described as negatively affecting the quality of TN communication [19]. The telephone nursing dialogue process brings structure to the call and aims to ensure high quality communication to form a basis for a correct and safe assessment and advice [14]. The telephone nursing dialogue process has been used clinically in Sweden for many years so there is extensive proven experience of the method, but as yet no scientific evidence exists to support it. The telephone nursing dialogue process has the potential to improve the quality and patient safety of TN, but studies are needed to determine whether there is sufficient scientific evidence to support the method.

### Research question

To describe the scientific basis of the phases of the telephone nursing dialogue process.

## The study

### Design

This study is an integrative literature review following the methodology described by Whittemore and Knafl [20]. The integrative review method enables the combination of data from diverse methodologies and is useful when the aim is to explore the evidence for nursing practice. The method includes problem identification, data collection, evaluation of data (quality appraisal), analysis and interpretation of data (data abstraction) and presentation of results ([20]). The Preferred Reporting Items for Systematic Reviews and Meta-Analyses (PRISMA) checklist [21]was used to guide the reporting of this study.

### Method

Selection criteria were predetermined before the search and formulated to gain a representative sample of articles representing a general TN context. Criteria of inclusion were peer-reviewed articles concerning nurse-provided TN, published in academic journals, available in full text and written in English or Swedish. Articles were excluded if they were editorials or expert opinion, concerned e-mail or video communication, communication between caregivers, telephone-provided follow-up for chronic conditions or inpatient visits, or TN as a part of nursing education. No time limit was applied since the telephone nursing research area only started to develop two decades ago.

A librarian was utilized to create the searches, and the searches were performed in the PubMed, CINAHL, Cochrane Database of Systematic Reviews and SwePub databases. Search terms were: Telephone nursing; Telephone advice; Telephone triage; Telephone communication; Teletriage; Telenursing; Telephone nurse*; and *Communication.* The literature search took place in August 2023, and a manual search was performed by screening reference lists and relevant literature in the field of telephone nursing. Grey literature was not included in the review.

**Fig. 2:**
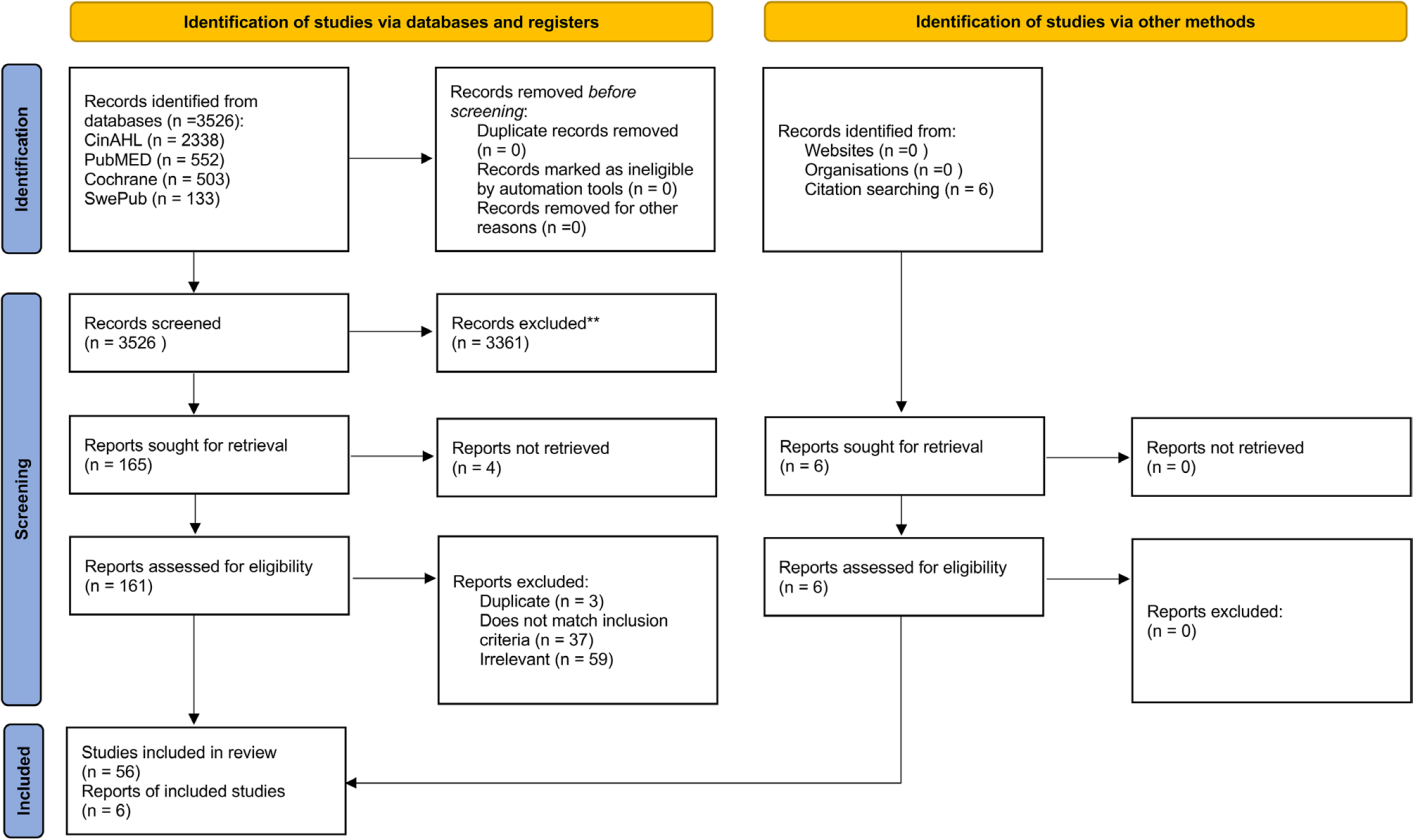
The search process

The systematic literature search process is described in Fig. 2. The literature search generated a total of 3526 articles. Articles were screened for adherence to selection criteria and relevance by both authors independently. Disagreement in assessment of relevance and selection criteria occurred for 19 articles, and these were discussed until a consensus was reached, leading to the inclusion of four of these articles. After exclusion of duplicates and irrelevant matches, a total of 62 articles were included in the review, 56 from the databases search, and six from the manual search. A summary of the included articles is presented in Table 1.

**Table 1:** Summary of included articles

Author (year). Country	Design	Approach	Sample	Focus
Berntsson et al. (2022). Sweden	Descriptive	Qualitative	12 nurses	Exploring nurses’ experiences and perceptions of patient safety when providing health advice over the phone
Björkman et al. (2018). Sweden	Descriptive	Qualitative	20 nurses	Nurses´ experiences of encountering callers with mental illnesses.
Björkman et al. (2019). Sweden	Descriptive	Qualitative	20 nurses	Describing the SHD and its features as a complex system.
Bonander (2007). Sweden	Descriptive	Qualitative	10 callers	Patients’ experiences of the caring relationship in telenursing
de Almeida Barbosa et al. (2016). Brazil	Literature review	Qualitative	10 studies	Identifying scientific evidence about the communication process in Telenursing
Derkx et al. (2009). The Netherlands	Quasi-Experimental	Quantitative	357 calls	Assessing the quality of communication skills of triagists, working at out-of-hours (OOH) centres
Engeltjes et al. (2023).	Descriptive	Qualitative	20 callers	Exploring patients’ experiences with obstetric telephone triage
Ericsson et al. (2019). Sweden	Descriptive	Qualitative	30 callers	The interaction between tele-nurses and callers with an evolving myocardial infarction
Eriksson et al. (2020). Sweden	Descriptive	Qualitative	19 nurses	Nurses’ strategies for managing difficult calls.
Ernesäter et al., (2012). Sweden	Descriptive	Qualitative and Quantitative	33 malpractice claims	Characteristics of malpractice claims following calls to the SHD during 2003–2010
Ernesäter et al., (2014). Sweden	Case-Control	Quantitative	33 malpractice claims and 26 matched control calls	Comparing communication patterns in calls subjected to a malpractice claim with matched controls
Ernesäter et al., (2016). Sweden	Mixed method	Qualitative and Quantitative	25 calls	Nurses’ communication and response to callers’ concern
Gamst-Jensen et al. (2017). Denmark	Mixed method	Qualitative and Quantitative	327 calls/19 patients	Communication patterns contributing to under-triage in a regional OOH service
Graversen et al. (2019). Denmark.	Instrument development	Quantitative	n/a	Development of an assessment tool assessing the quality of communication, patient safety and efficiency of telephone triage
Graversen et al. (2020). Denmark	Quasi-Experimental	Quantitative	1294 calls	Quality of communication in telephone triage
Greenberg (2009). United States	Descriptive	Qualitative	10 nurses	Development of a theoretical model of the process of telephone nursing
Gustafsson et al. (2018). Sweden	Descriptive	Qualitative	10 callers	Describing callers’ needs for reassurance when receiving self-care advice for minor illnesses
Gustafsson et al. (2020). Sweden	Descriptive	Qualitative	123 callers	Callers’ experiences and perceptions of satisfaction with telephone nursing.
Holmström & Höglund (2007). Sweden	Descriptive	Qualitative	12 nurses	Ethical dilemmas in telephone nursing
Holmström et al. (2016). Sweden	Descriptive	Qualitative	10 callers	Older persons’ experiences of telephone advice nursing
Holmström et al. (2017). Sweden	Descriptive	Qualitative	10 nurses	Nurses’ experiences of frequent callers to primary healthcare centres
Holmström et al. (2020). Sweden	Descriptive	Qualitative	24 nurses	Nurses’ experiences of using a clinical decision support system for triage of emergency calls
Holmström et al. (2022). Sweden	Descriptive	Qualitative	24 nurses	Nurses’ strategies for handling difficult calls to emergency medical dispatch centres
Johnson et al. (2015). Sweden	Instrument development	Quantitative	n/a	Developing a self-assessment tool aiming to raise nurses’ awareness of their communication and interpersonal competence
Jones et al. (2012). Australia	Literature review	Qualitative	Unknown	Exploring the dimensions of caring in the telenursing environment
Kaminsky et al. (2009). Sweden	Descriptive	Qualitative	12 nurses	Nurses’ understanding of their work
Kaminsky et al. (2013). Sweden	Descriptive	Qualitative	21 callers	Parents’ expectations and experiences of telenursing regarding paediatric health issues
Kaminsky et al. (2014). Sweden	Descriptive	Qualitative	23 managers	Managers perceptions’ of the goals of telephone nursing work
Kaminsky et al. (2017). Sweden	Literature review	Qualitative	24 studies	Providing a comprehensive understanding of telephone nursing in Sweden
Kaminsky et al. (2020). Sweden	Descriptive	Qualitative	12 nurses	Nurses´ views on telephone nursing for patients with respiratory tract infections in primary healthcare
Kvilén Eriksson et al. (2015). Sweden	Descriptive	Qualitative	10 callers	Parents’ experiences of monitoring calls in children with gastroenteritis
Larsson et al. (2022). Sweden	Descriptive	Qualitative	11 nurses	Nurses’ experiences of managing calls with patients affected by mental illness in primary healthcare
Landqvist(2011). Sweden	Descriptive	Qualitative	n/a	The professional use of feedback signals in medical advice calls
Leppänen (2010). Sweden	Descriptive	Qualitative	276 calls / 18 nurses	Power in social interaction between nurses and callers in telenursing
Lopriore et al. (2017). Australia	Descriptive	Qualitative	196 calls	Exploring how healthcare is delivered over the telephone
Lopriore et al. (2019). Australia	Descriptive	Qualitative	196 calls	Exploring the accomplishment of physical examination on a health helpline.
Mattisson et al. (2023). Sweden	Instrument development	Quantitative	616 callers	Psychometric evaluation of the Telenursing Interaction and Satisfaction Scale (TISS)
Mattisson et al. (2023). Sweden	Descriptive	Quantitative	466 callers	Role of interaction for caller satisfaction in telenursing
Morgan & Muskett (2020). United Kingdom	Descriptive	Qualitative	Unknown	Interactional misalignment in the UK NHS 111 healthcare telephone triage service
Murdoch et al. (2014). United Kingdom	Descriptive	Qualitative	51 calls	Comparing doctors and nurses communication with patients in primary care telephone triage consultations.
Murdoch et al. (2015). United Kingdom	Descriptive	Qualitative	22 calls	The impact of using computer decision-support software in primary care nurse-led telephone triage
Nagel & Penner (2016). Canada	Literature review	Qualitative	8 studies	Conceptualizing Telehealth in Nursing Practice
Pettinari & Jessop (2001). United Kingdom	Descriptive	Qualitative	12 nurses	Nurses’ perceptions of interactional practices to manage the absence of visual cues in telephone nursing
Purc-Stephenson & Thrasher (2010). Canada	Literature review	Qualitative	16 studies	Nurses’ experiences with telephone triage and advice and factors that facilitate or impede their decision-making process
Rysst Gustafsson & Eriksson (2020). Sweden	Literature review	Qualitative	30 studies	Factors that indicate quality in telephone nursing
Röing & Holmström (2015). Sweden	Descriptive	Qualitative	6 nurses/5 managers	Malpractice Claims in Swedish Telenursing
Röing et al. (2013). Sweden	Descriptive	Qualitative	6 nurses/121 calls	Threats to patient safety in Nurses’ dialogues with callers
Sandelius & Wahlberg (2019). Sweden	Descriptive	Qualitative	19 nurses	Nurses’ experiences of monitoring calls to parents of children with gastroenteritis
Sands et al. (2013). Australia	Descriptive	Qualitative	197 calls	to identify and articulate how mental health telephone triage clinicians manage psychiatric crisis and emergency via the telephone
Skogevall et al. (2020). Sweden	Descriptive	Qualitative	199 nurses	Telephone nurses’ experiences in their encounters with frequent callers
Smits et al. (2017). The Netherlands	Instrument development	Quantitative	114 calls	Development of an instrument to assess the quality of telephone triage in out-of-hours primary care services
Snelgrove (2009). United Kingdom	Descriptive	Qualitative	13 nurses	The ways nurses construct a nursing identity and shape their work in a call-centre environment
Ström et al. (2006). Sweden	Descriptive	Qualitative	12 nurses	Nurses’ perceptions of providing advice via a telephone care line
Ström et al. (2009). Sweden	Descriptive	Qualitative	12 callers	Callers’ perceptions of receiving advice via a medical care help line
Wahlberg & Wredling (2001). Sweden	Descriptive	Qualitative and Quantitative	144 callers	Callers’ experiences of telephone advice nursing
Wahlberg et al. (2003). Sweden	Descriptive	Qualitative and Quantitative	25 nurses	Telephone nurses’ experience of problems with telephone advice in Sweden
Wahlberg et al. (2005). Sweden	Descriptive	Qualitative	7 nurses	Exploring what telephone nurses base their assessments on
Valanis et al. (2007). United States	Descriptive	Quantitative	1782 callers	Aspects of the advice call process and predictors of caller follow-through
Vilstrup et al. (2019). Denmark	Descriptive	Quantitative	200 calls	Comparing communicative parameters in general practitioner led and nurse led telephone triage
Wouters et al. (2020). The Netherlands	Descriptive	Qualitative	24 nurses	Nurses’ clinical reasoning and decision-making during conversations with callers suspected of having acute cardiac events
Wärdig et al. (2022). Sweden	Descriptive	Qualitative	15 nurses	Nurses’ experiences of suicide risk assessment in telenursing
Yliluoma & Palonen (2019). Finland	Descriptive	Qualitative	9 nurses	Nurses’ experiences of interaction with patients and family members

The included articles were carefully read and both authors independently assessed the quality and risk of bias of the articles using standardized protocols for quality appraisal from the Joanna Briggs Institute (JBI) [22]. Due to the heterogeneity of studies included, the protocol used for quality appraisal depended on the study design. Protocols used were Checklist for Analytical Cross-Sectional Studies, Checklist for Case Control Studies, Checklist for Qualitative Research, and Checklist for Quasi-Experimental Studies. A suitable protocol for validation studies did not exist from JBI. Therefore, the QUAVALS protocol [23] was used to assess quality for studies with a validation design. The results from the quality appraisal from both authors were then compared and no disagreements were found. All articles were considered suitable for inclusion in the review.

### Analysis

The process of data analysis in the integrative review consists of data reduction, data display, data comparison, and conclusion drawing and verification. The data reduction phase involves the determination of an overall classification system for working with the data, as well as extracting and coding data from primary sources [20]. The classification system features the five phases of the telephone nursing dialogue process (i.e. *Opening, Listening, Analyzing, Motivating*, and *Ending*). The classification system was extended with keywords derived from the SHD descriptions of the content of each phase. As an example, the keywords for the phase *Opening* were “welcoming, tone of voice, showing interest, dedication, encouragement, wanting to help” and the content in the textual unit should be in the context of starting the call. The first step of the analysis was thus deductive, and textual units containing content relating to the phases of the telephone nursing dialogue process were extracted. A matrix matching the five phases of the telephone nursing dialogue process was constructed in Microsoft Word, and this matrix was used during the extraction and sorting of data.

The data display phase involves moving and sorting the extracted data into a display that compiles the data from the different sources [20]. Data from review articles were manually reviewed to avoid reporting biases, and textual units from reviews that built on original studies already included in our review were excluded (n = 4). All remaining textual units were then sorted into the matrix depending on what phase the textual unit described. The data extraction and deductive sorting was done by both authors separately, and discrepancies in interpretation were resolved through discussion between the authors until a consensus was reached.

When all textual units were sorted depending on phase, the analysis entered the data comparison phase. This is when the data are examined to identify patterns in the content [20]. In this step, the analysis proceeded with an inductive approach. All data describing each phase of the telephone nursing dialogue process were transferred from the matrix in Microsoft Word to an online computerized software called QCAmap [24]. An iterative process of categorization was performed in the software. Similar variables – or textual units in our case – were grouped together based on similarity in content. This enabled the formation of categories that further contributed to the understanding of the phases of the telephone nursing dialogue process. The categories also contributed to a deeper and more detailed description of the content of each individual phase. The analysis in QCAmap was done separately for each phase of the telephone nursing dialogue process. This allowed us to enter the final phase of data analysis, where data are synthesised and conclusions are drawn. The results of the analysis are also verified from primary sources [20]. A narrative synthesis was chosen due to the heterogeneity of the included studies regarding design and perspective. A narrative synthesis implies that data from both qualitative and quantitative studies are combined. We went back to the descriptions of the telephone nursing dialogue process to verify that the categories created for each phase fitted well into the descriptions of the content of the phases as presented in the method description [14].

### Ethics

Ethical approval was not required for this literature review.

## Results

### Description of studies

Sixty-two articles matching the selection criteria were included in the review. An overview of article design, approach, aim and sample is presented in Table 1. From the 62 articles included, 47 studies had a descriptive design, six were literature reviews, four were instrument development studies, two were quasi-experimental studies, two were mixed method studies, while one was a case-control study. Forty-seven articles had a qualitative approach, ten articles had a quantitative approach, and five articles combined both quantitative and qualitative approaches. The sample sizes are described in Table 1. The 62 included studies were conducted in Sweden (n = 39), the United Kingdom (n = 5), Denmark (n = 4), Australia (n = 4), the Netherlands (n = 4), the United States (n = 2), Canada (n = 2), Brazil (n = 1) and Finland (n = 1). The number of articles that provided data for the different phases of the telephone nursing dialogue process were as follows: Opening (n = 32), Listening (n = 45), Analysing (n = 50), Motivating (n = 48), and Ending (n = 35). The result of the data analysis is presented in Tables 2 and 3.

**Table 2:** Matrix of the articles providing data for each phase

Article		Opening	Listening	Analyzing	Motivating	Ending
Berntsson et al. (2022).		X	X	X		
Björkman & Salzmann-Erikson (2019).			X		X	
Björkman & Salzmann-Erikson (2018).		X	X	X	X	X
Bonander (2007).		X	X	X	X	X
de Almeida Barbosa et al. (2016).			X	X		
Derkx et al. (2009).			X	X		
Engeltjes et al. (2023).		X	X	X	X	X
Ericsson et al. (2019).			X	X	X	
Eriksson et al. (2020).		X	X	X	X	X
Ernesäter et al., (2014).				X		X
Ernesäter et al., (2012).				X	X	X
Ernesäter et al., (2016).				X	X	
Gamst-Jensen et al. (2017).		X	X		X	X
Graversen et al. (2020).			X	X		X
Graversen et al. (2019).		X	X	X	X	X
Greenberg (2009).		X	X	X	X	X
Gustafsson et al. (2018).		X	X	X	X	X
Gustafsson et al. (2020).			X		X	X
Holmström & Höglund (2007).				X	X	
Holmström et al. (2016).		X	X	X	X	X
Holmström et al. (2017).			X		X	
Holmström et al. (2020).				X	X	
Holmström et al. (2022).			X	X	X	X
Johnson et al. (2015).		X	X		X	X
Jones et al. (2012).		X	X	X	X	X
Kaminsky et al. (2009).		X	X	X	X	X
Kaminsky et al. (2013).		X	X	X	X	X
Kaminsky et al. (2014).		X		X	X	X
Kaminsky et al. (2017).				X	X	X
Kaminsky et al. (2020).			X	X	X	X
Kvilén Eriksson et al. (2015).		X	X		X	X
Larsson et al. (2022)		X	X	X	X	
Landqvist(2011).		X	X			
Leppänen (2010).			X	X	X	X
Lopriore et al. (2017).		X	X	X		
Lopriore et al. (2019).				X		
Mattisson et al. (2023).		X	X	X	X	X
Mattisson et al. (2023).		X	X	X	X	X
Morgan & Muskett (2020).		X	X	X	X	X
Murdoch et al. (2014).				X		
Murdoch et al. (2015).				X		
Nagel & Penner (2016).		X	X	X	X	
Pettinari & Jessop (2001).		X	X	X	X	
Purc-Stephenson & Thrasher (2010).			X	X	X	X
Rysst Gustafsson & Eriksson (2020).		X	X	X	X	X
Röing & Holmström (2015).				X		X
Röing et al. (2013).				X	X	X
Sandelius & Wahlberg (2019).					X	X
Sands et al. (2013).		X	X	X	X	
Skogevall et al. (2020).			X		X	
Smits et al. (2017).		X	X	X	X	X
Snelgrove (2009).		X	X	X	X	
Ström et al. (2006).			X	X	X	X
Ström et al. (2009).		X	X		X	
Wahlberg & Wredling (2001).			X		X	
Wahlberg et al. (2003).		X	X	X	X	
Wahlberg et al. (2005).				X		X
Valanis et al. (2007).			X		X	
Vilstrup et al. (2019).			X	X		
Wouters et al. (2020).				X		
Wärdig et al. (2022).		X		X	X	
Yliluoma & Palonen (2019).		X		X	X	X
**TOTAL**		**32**	**45**	**50**	**48**	**35**

**Table 3:** Categories

Phase	Categories
Opening	Welcoming the caller
	Establishing a caring relationship
Listening	Inviting the caller to tell their story
	Active listening
	Confirming understanding
Analysing	Gathering information
	Assessing information
	Verifying and clarifying information
Motivating	Reaching a final assessment
	Informing the caller
	Giving advice and guidance
	Creating mutual agreement and understanding
	Supporting the caller
Ending	Checking for mutual agreement and understanding
	Giving safety-net advice
	Monitoring the caller
	Rounding off

### Opening

The result reveals that the nurse typically opens the call with a greeting and a presentation. Receiving the caller in a welcoming and respectful way lays the foundation for establishing a caring relationship. Opening sequences are typically short and quickly transition into the next phase of the call by eliciting the reason for calling [25, 26].

#### Welcoming the caller

The nurse starts by greeting the caller and introducing him/herself by name and title [2, 25–27] and gathers personal details [28–30]. The caller should be greeted in a friendly and respectful manner to facilitate connection with the caller [6, 19, 31–39]. This implies that the nurse uses a friendly tone of voice [2, 27, 30–32], is present and listening [2, 19, 32, 34, 40], remains calm and understanding [19, 32, 34–36, 38–40] and displays a willingness to help [31, 41]. The tone of voice is central to conveying emotional status and mood, and voice mode together with support signals indicates that the nurse is interested, engaged and willing to help [42, 43]. The voice is utilised as a communication tool by adjusting the rhythm and tone to match the situation [37, 41].

The opposite of a welcoming and friendly reception is described when the nurse has a superior attitude, and when the nurse expresses feelings of irritation, condescension, patronisation, or disagreement or distrust about the magnitude of the problem [19, 31, 44]. This can lead to the caller having feelings of being inconvenient, worthless, dismissed and evicted [31, 35, 44], thus inhibiting the willingness to share important information about their symptoms [2].

#### Establishing a caring relationship

Forming an alliance with the caller is important for establishing trust and confidence so that the caller feels free to present the problem and reason for calling [2, 5, 25, 26, 30, 31, 34, 38, 39, 45–51]. The first impression is crucial for gaining the caller’s confidence, and trust and confidence are easily lost if the nurse is stressed or behaves in an unprofessional way [31, 45, 46, 49]. A caring relationship facilitates the delivery of information later in the conversation, favouring the assessment and enhancing further healthcare contacts [26, 31, 34, 45, 46, 48]. To establish a relationship, the nurse should create a sense of presence in the conversation [37, 52]. Presence can also be demonstrated through a calm and friendly composure [31, 32, 47] and the caller should be the centre of attention [46]. Personalizing a call can be done by addressing the caller by name [47, 49], by the use of humour (when appropriate) or by alluding to something that the nurse and caller have in common [31, 34, 37].

### Listening

When the nurse has opened the call, the call quickly proceeds to the listening phase. The nurse invites the patient to tell their story, listens actively and confirms his or her understanding with the caller.

#### Inviting the caller to tell their story

The nurse starts this phase by asking an open and nonspecific question to invite the caller to tell their story [2, 25–27]. The caller usually presents their reason for calling as a narrative [25] and prefers to tell the story at the beginning of the call [53] It is important that the caller be allowed to tell their story without being interrupted by questions or unnecessary remarks [29, 43, 47, 53, 54]. The nurse invites the caller to tell their story and present their problems using their own words [25, 26, 29, 43, 50, 53–56]. The nurse steers the call by assigning the caller the role of speaker, with the nurse playing the part of listener [43, 57].

The caller is given time and space to speak [28–30, 33, 40, 45, 51, 53]. The nurse is active and responsive in listening [38, 39, 43, 58]. One way of doing this is to use support signals, like “OK?”. “Mhm” and “Yes?”. Support signals are also used to steer the conversation and to change focus, regain focus, or end topics [43], for instance, when the caller’s story loses focus [53, 59]. The conversation with the nurse holds the possibility for the caller to share worries and concerns [6, 34], and listening has a calming effect [43, 56]. When the nurse remains calm while listening, this contributes to relieving distress and worry in the caller [30, 40, 47, 60]. Blocking or discouraging the caller from telling their story is dangerous and imposes a risk of undertriage [44].

It is essential that the nurse is aware of the caller’s exposed position and shows respect by placing the caller at the centre of attention [46], taking the caller seriously [6, 31, 35, 46], respecting each person as unique [31, 46, 47] and confirming the caller’s emotions about the concern [31]. The nurse shows interest in the patient’s story, allows them to speak without interruption and is genuine, sensitive and responsive [31, 32, 38, 39, 43, 53, 61–64]. Showing interest signals a will to understand [43] beyond the purely medical aspects of the caller’s situation [31], as well as a will to help [65]. One way to show respect is to adopt an attitude of humility and to avoid a superior approach [43, 46, 61]. The caller should not be interrupted, trivialized or disrespected. Being disrespected creates distrust in the service, increases the need for second opinions and visits to emergency services [47] and lowers the quality of the TN service [19]. Instead, the nurse should partner with the patient, striving for mutual respect and trust through dialogue [5, 31, 34, 43].

#### Active listening

While the caller is telling their story, the nurse adopts an active listening position where the caller’s story is in focus [29, 42, 43, 50, 52, 56–58, 66]. Active listening is expressed by support signals that convey interest and openness to continued interaction. Active listening facilitates the establishment of a caring relationship, builds confidence, relieves anxiety and provides a quicker basis for assessment [43]. The nurse expresses empathy and caring [27, 28, 30, 34, 42, 43, 59], acknowledging the caller´s feelings and experiences [42, 50]. Empathy is typically communicated when the caller expresses suffering or distress [43]. Empathy is expressed through the choice of words, voice, intonation [27] or support signals and projects compassion, warmth and an identification with the speaker’s emotions [43].

While listening actively to the caller, the nurse tunes in to the caller’s story and listens to both verbal and nonverbal clues [49, 53]. Tuning in implies trying to take in the situation and the caller to reach a comprehensive understanding of the situation [2, 7, 32, 43, 53].

#### Confirming understanding

When the caller has shared their story with the nurse, the nurse confirms the caller [27, 28, 35, 42] to convey both an understanding of the situation [32, 35, 43] as well as the caller’s feelings and experiences [35, 43]. The nurse remains calm and listens to the caller and then summarizes what the caller has said to ensure that the information has been registered and understood correctly [32]. Confirmation can be given using support signals and by being attentive to the caller’s feelings and acknowledging or naming these feelings in words [27, 28, 42, 46]. The use of support signals is important, since they can also be non-confirmative, signalling that the listener is bored, uncomprehending, and sceptical, thus blocking communication and disrespecting the caller. The nurse’s response to emotions by using support signals reflects the nurse’s attitudes and reactions [43].

### Analyzing

When the caller has finished their narrative, the nurse advances to the next stage in the process and starts asking questions. In this phase, the nurse gathers and assesses information, and verifies with the caller that the information has been understood correctly.

#### Gathering information

Asking the right questions is essential to gather the information that is needed to make an accurate assessment [2, 5, 17–19, 25, 28–30, 32, 33, 36, 38–41, 45, 49, 58, 62, 67–69]. The nurse will in some cases open this phase of the call by explaining to the caller that she will now proceed with a series of questions to aid the assessment [26, 49]. The nurse gathers information by asking questions about the caller’s perception of the problem [36, 55], physical symptoms [2, 17, 18, 33, 49, 57, 68, 70], vital signs [25, 52] previous medical history [18, 28, 33], medications [28, 33], the caller’s situation [49, 52, 58, 70] and/or emotional state [41].

The nurse gathers increasingly specific information about the health needs and context of the caller [2]. Open-ended questions have been found to provide the nurse with more information compared to closed questions [18, 45, 71]without increasing the total time of the calls [18, 67, 72]. However, closed questions are also necessary in some cases when the nurse is gathering specific information to pinpoint the problem [2, 33]. Clarifying statements and declarative (yes/no) questions can also be used to specify the concern and to rule out other diseases or conditions [2]. Some symptoms require detailed questioning, rephrasing questions with examples, or asking the caller to perform self-tests to enable the nurse to gain an overview of the situation and visualize the caller and the symptoms [36, 49, 58]. The caller can be instructed to perform physical examinations like capillary refill time or measuring the fever, to perform a certain action to know if it provokes pain or not, or to say whether bodily features appear “normal” [37, 47, 49, 58, 73].

The absence of visual cues implies that good questioning skills are needed to gather information that would normally be observed with the naked eye. It also requires that the nurse listens attentively and interprets nonverbal clues and background sounds, such as breathing, tone of voice, word choice, dissonances and paralanguage to build an understanding of the patient and the situation as a whole [5, 19, 29, 37, 41, 49, 51, 58, 59, 62, 69, 70, 74]. The information should be gathered with a holistic approach, aiming to understand the caller’s context and situation so that information about the physical, emotional or social impact of the problem is obtained [18, 34, 50, 58, 66, 75]. Information about the caller’s abilities and context can support decision-making [37, 70].

Nurses also gather information through medical records [2, 29, 37] and by using Computerized Decision-Support Systems (CDSS) [29, 69, 76]. Nurses using CDSS ask more declarative questions, which can make it easier to rule out a variety of medical problems. But declarative questions can also impose constraints on the topical agenda and steer the conversation in the wrong direction [68, 77]. Nurses are responsible for leading and structuring the call [33, 37, 48, 59, 74]. Structuring the call enables the nurse to obtain correct information [19] and obtain a distinct interaction that is concise and advancing forward [32, 34]. It is important that the nurse strives to gather first-hand information, thus avoiding talking through a third party [18, 19, 33, 36, 47, 78]. Second-hand information can impose risks to patient safety in terms of faulty or misleading information [19, 36, 71], as well as ethical dilemmas [78]. Asking questions has been described as detective work [5], and the nurse has only the information provided by the caller to rely on [78]. The nurse must believe in the caller [34] but at the same time not simply accept the caller´s opinion about the cause of the problem, since this in some cases can be misleading and cause safety threats [72].

#### Assessing information

A central part of TN is to make an accurate medical assessment of symptom severity and urgency [28, 48, 62]. The assessment is based on detailed information about physical signs and symptoms, as well as the caller´s context, [37, 52, 60] and nonverbal cues [5, 19, 37, 49, 58, 69, 70, 74]. To make a correct assessment, the nurse needs to identify and uncover potential medical problems that can pose a risk to the caller´s health and act appropriately [28, 33]. The nurse strives to build an overview and a mental image of the caller and the situation, and to gain a sense of the context [2, 37, 52] to identify the caller’s needs [46, 47]. This implies that the nurse attempts to construct a mental image of the caller and context and to visualize the situation to compensate for the lack of visual data [49, 52, 58, 70]. To identify the symptom location, nurses will in some cases touch the location on their own body while simultaneously verifying that the location corresponds to that of the callers. This is described as a technique for visualizing body location [49].

Nursing skill, knowledge and experience aid assessments and decision-making and allows the nurse to interpret and assess the callers’ condition over the telephone [2, 34, 52, 58, 62, 70, 71]. When unsure about the assessment, nurses often turn to colleagues, such as nurses and physicians, for information and advice [2, 58]. Protocols and CDSS are also used to guide information-gathering, aid assessment and support decision-making [2, 18, 28, 32, 58, 76, 77]. However, the CDSS response options do not always match the caller’s report of their symptoms [2, 77], which can lead to a dilemma if the CDSS recommendation does not align with the nurse’s clinical reasoning [70]. In these cases, nurses will sometimes rely on their experience and knowledge, and overrule the CDSS recommendation [76]. According to Snelgrove [34], the software should be treated “*as an adjunct rather than an equal partner in the decision-making process*” (p.359).

#### Verifying and clarifying information

The inability to see the caller in person can cause uncertainty about the assessment [19, 75], especially in the presence of language barriers [36, 75] or imprecise or vague communication [57]. Sometimes the nurse can perceive the caller is giving too limited or conflicting information or suspects that the caller is understating or overstating the magnitude of the problem [29, 36, 70]. One way of dealing with this uncertainty is to verify and clarify information with the caller [2, 37]. Information can be verified or clarified by asking for confirmation [54, 58], summarizing [29, 32, 33, 42, 54], repeating [18, 29, 54], or comparing [2, 25].

Asking for confirmation implies that the nurse asks questions to verify that the information gathered is correct [54, 58]. Rephrasing involves rephrasing questions or information [49]. Summarizing involves summarizing what the caller has said or asking the caller to summarize what they have understood to verify that information has been understood correctly [32, 54, 55]. Repeating involves repeating information in the form of clarifying statements or questions [18, 42, 59]. Comparing involves asking a series of questions and comparing incoming information with existing nursing knowledge and experience to rule out conditions [2, 25, 68]. Such questions are typically posed in the form of negative declaratives to rule out, rather than to confirm the presence of these signs [25, 68]. These declarations are often prefaced with ‘And’ as in *“…And you don’t have any fever?”* [68].

### Motivating

In the next phase of the telephone nursing dialogue process, the nurse reaches a final assessment, gives advice and guidance, and informs the caller. The nurse strives to achieve a mutual understanding of the problem and the assessment, and a mutual agreement about the plan of action. The nurse also supports the caller using confirming and empowering strategies.

#### Reaching a final assessment

When information is appropriately gathered, assessed, and verified, the nurse reaches a final assessment that includes a triage decision about what advice to give the caller and which care level to recommend [2, 5, 35]. The triage decision concerns the output of the call [2, 27, 33, 34, 36] and the output of the call are the nursing actions designed to solve problems [42] and meet the caller’s needs [2]. The output can include providing information, giving self-care advice and/or further referring the caller [2, 5, 35]. The nurse assesses the caller’s capabilities of performing self-care and managing the situation [5, 44]. The plan of action is based on the final assessment and tailored to the urgency or acuity of the problem, taking into consideration the caller’s situation and capabilities of managing the situation, performing self-care, available resources, and access to healthcare [2, 5, 33, 44, 50, 52]. The nurse´s final assessment is presented to the caller [26].

#### Informing the caller

The nurse informs the caller through the provision of information and explanation. The nurse explains bodily functions [5, 61] and the reasoning behind the triage decision [5, 31, 49]. The nurse gives information about symptoms and risks [6, 38, 39], as well as medications and potential side effects [7]. The information should be clear, correct, credible [7, 19, 28, 30, 35, 49, 56, 64] and relevant to the caller’s situation [35, 38, 39] as too much information might cause stress and confusion [19, 72]. Informing the caller creates an awareness about the underlying cause of the condition [45, 78] and has the potential to inspire the caller to think in new ways about the situation [32].

#### Giving advice and guidance

Depending on the final assessment, the nurse advises the caller on what to do and guides the caller to the correct level of care [5–7, 35, 36, 38, 39, 41, 48, 65, 67]. The advice is adjusted and adapted according to the caller’s specific situation and needs [2, 27, 32, 33, 35, 42, 44, 58, 63]. The advice may apply to the care, management and/or treatment of the medical problem [6, 7, 26, 67], self-care actions [6, 35, 36, 56],72) or referral to the correct level of care [5, 7, 28, 35, 48, 65]. The advice should be practical, clear and hands-on [7, 42, 44], personalized to match the caller’s needs [7, 65], and correct and evidence-based [65]. The advice should be clear, easy to understand and easy to follow [33, 35, 48], as overly complex advice makes it difficult for the caller to focus [44]. According to Leppänen [60], explicit advice is used to increase the caller’s compliance, for instance, when the medical problem is potentially very serious or urgent.

The nurse gives relevant self-care advice to enable the caller to be self-reliant and to manage the situation correctly [5, 28, 32, 33, 75]. Advice on self-care actions is appreciated since many callers want to avoid unnecessary visits to the healthcare facility [6, 65] or taking unnecessary antibiotics [62]. If the medical problem warrants further examination or treatment, the nurse guides the caller to the correct caregiver and level of care [7, 45, 48, 50, 56, 74]. The CDSS can aid the assessments and recommendations [58, 76] and reduces professional vulnerability and the possibility of legal consequences in case of erroneous assessments or advice [58].

#### Creating mutual agreement and understanding

The nurse attempts to achieve a mutual understanding of the problem and the solution [27, 38, 39, 42, 44, 52, 57, 61]. The nurse adapts language, voice mode and/or speech rate depending on the age, fluency and level of education of the caller [33, 37, 46, 49, 59] follows up on the caller’s understanding to ensure that the triage decision and the advice are understood and feasible [19, 33, 36, 67, 74]. The nurse checks that she has understood the caller correctly by summarizing the triage decision and advice. The nurse verifies the summary with the caller and adjusts the summary if necessary [19, 28, 32, 40, 67].

A plan of action is established together with the caller [27, 44, 52]. Acceptance of and compliance with the plan of action increases when the caller takes an active part in the decision-making [35, 38, 39]. The nurse and caller work together to explore options and find solutions that the caller is satisfied with and feels secure with [2, 35, 48, 50, 64]. The caller´s free will is respected [19, 26, 33, 37, 63].

Sometimes there may be disagreement regarding the triage decision, for example, when there is a discrepancy between expectations of care and assessment of care needs. Ensuring mutual understanding and agreement require pedagogical competence and an ability to explain what actions are medically justified [61]. If the nurse fails to achieve a mutual agreement about call output, the caller might feel dismissed and unsafe [31, 65] which can lead to frustration, dissatisfaction and not following the triage decision [19, 61]. The nurse should bear in mind that there is a possibility that self-care advice might be perceived as gatekeeping intended to hinder access to physical care facilities [48]. It is essential that the nurse is respectful towards the caller [31], which implies talking in a calm voice and tone, and being genuine, caring, compassionate, friendly, helpful and patient [31, 35, 52, 56, 65]. Having a nonchalant attitude, diminishing the caller’s concern or providing unsatisfactory explanations manifests a lack of respect [31, 46] and can result in strong reactions, anger and irritation [46]. A mutual understanding and a respectful treatment inspire trust in the nurse and the call output [46, 65].

#### Supporting the caller

The nurse supports the caller to empower them in coping with the illness [38, 39, 42, 51, 57, 65, 79]. The support can consist of positive feedback that the self-care performed is correct, a calm appearance and emotional confirmation, as well as confirmation of the caller’s own decision to seek care [5, 6, 35, 37, 57, 59, 65, 79]. Receiving personalized support based on the care seeker’s knowledge and personal circumstances can lead to calm, reassurance and satisfaction of needs [33, 45, 47, 57, 65]. Many callers express concern, and the nurse responds with an ambition to inspire comfort and calm [17, 48, 59, 61, 67]. This can imply calming the caller with information that the problems are transient and describing the natural course of the illness [19, 32]. Nurses confirm and empower the caller using supportive strategies like offering reassurance, encouragement, acknowledgement, and hope [2, 41, 42, 44, 50, 51, 58, 61].

### Ending

Before ending the call, the nurse checks for mutual agreement and understanding, provides support and safety-netting advice, follows up the call if needed and ultimately rounds off the call.

#### Checking for mutual agreement and understanding

Before ending the call, the nurse needs to check for mutual understanding and agreement [2, 19, 31–33, 38, 39, 55, 79]. This can be done by repeating, paraphrasing or summarizing what has been agreed upon [2, 17, 31–33, 42, 55] or by asking the caller to repeat what has been decided to verify the caller´s understanding and to crosscheck that the follow-up action is understood and feasible [18, 19, 28, 79]. The nurse should verify that the caller is comfortable with the choice of intervention [2, 19, 28, 48, 60] either by observing the caller´s responses to the advice given [60] or by asking the caller directly [2, 19, 28, 47]. The call should be terminated with a clear agreement on further handling of the situation [2, 19, 28, 31, 37, 44].

#### Giving safety-net advice

Before terminating the call, the nurse should give safety-net advice about warning signs and symptoms that warrant a new assessment, or who to contact in case the symptoms worsen or if there is no improvement [2, 7, 26–28, 30, 33, 47, 58, 62, 74]. The caller should be invited to call again in case the symptoms worsen or show no signs of improvement [31, 32, 40, 62]. Nurses also sometimes use the strategy of “over triaging” as a form of safety netting [59].

#### Monitoring the caller

In some cases, a monitoring call (also called a follow-up call) could be necessary. Monitoring the caller may be indicated to observe the course of the disease and to safeguard the initial assessment, especially if there remains some uncertainty regarding the assessment or the choice of intervention [2, 5, 6, 19, 46, 57, 69, 72, 79]. A monitoring call provides an opportunity to re-assess the situation, to ask more questions and give new advice [2, 5, 6, 46, 57, 69, 72, 79]. A monitoring call may result in a deeper, more personal contact as the caller feels that their situation is being taken seriously, that they are secure and cared for, and that they are not alone [2, 5, 6, 32, 57, 79]. As such, this call has the potential to ease worry. It can also give the caller a feeling of shared responsibility with the nurse [6].

A monitoring call can provide output validation about whether the initial assessment and advice were appropriate and thus constitute a learning opportunity for the nurse [2, 58, 75]. It also enables the caller to safely perform self-care at home [6, 79], thus avoiding unnecessary visits and relieving the pressure on healthcare [79]. The caller’s self-care ability could also be strengthened through a mutual evaluation of self-care effect and symptom development during the monitoring call [32, 42, 79].

#### Rounding off

Ultimately, the nurse rounds off the call. This is typically done through an increase in speech pace of the nurse, less participation in the conversation from the caller and using closing questions [26]. These communicative features mark the end of the conversation. However, it is important that the nurse ties together the conversation in a meaningful way before ending the call [31] and that the pressure of calls waiting does not lead to a premature closing of the call [71].

## Discussion

The aim of the study was to describe the scientific basis of the phases of the telephone nursing dialogue process, and 62 articles were found with each supporting at least one of the phases. All five phases in the telephone nursing dialogue process were supported by a range of articles (n = 32–50), and the phases could therefore be described as having a sound evidence base.

We found 32 articles describing the *Opening* of the call. The literature described the importance of opening the call with a greeting and presentation, and to establish a caring relationship. This corresponds well to the methodological description of this phase [14]. Both the result and the methodological description describe that the voice is used as a tool to signal interest and to initiate the dialogue. Nurses use their voice to convey empathy, confidence and emotional support, and the use of the voice can influence the caller’s opinions about the attitude, knowledge, and caring qualities of the nurse [80]. These unspoken signals are referred to as paralanguage, and enables the identification of feelings such as anger, contempt, or doubt [54]. There is always a risk that trust is lost if the voice signals to the caller a lack of interest or an unwillingness to help.

The data on the *Listening* phase consist of textual units identified in 45 scientific articles and align with the methodological description by Runius [14] as well as other descriptions of patient-centred communication [81, 82]. Listening is the foundation of meaningful relationships and involves more than just hearing the patient. It is a deliberate act of paying attention to the speaker that requires presence and a conscious effort to search for meaning and understanding [82]. Active listening requires a response to the caller’s narrative, which could be accomplished by both support signals [43, 83, 84], or summarizing [81]. The telephone nursing dialogue process has been criticized for providing relatively vague instructions on the use of support signals [84]. Robertson [85] recommends using a minimum of verbal encouragers, perhaps because a frequent use of support signals might rush the caller’s story forward or interrupt the caller. Due to individual or cultural variations, is might therefore be difficult to provide universal instructions on how and when to use support signals. Rather the nurse’s sensitivity as to when support signals are appropriate could be more important.

A total of 50 articles provided data on the *Analyzing* phase of the call. The data are well aligned with the methodological description of the telephone nursing dialogue process [14]. To reach a correct assessment, the nurse needs to interpret both verbal information and nonverbal cues such as tone of voice and background sounds. The telephone nursing dialogue process has been criticized for not providing clear instructions on how this interpretation is to be performed [84]. The telephone nursing dialogue process thus encounters the familiar problem of describing the process of interpretation. According to Corey [86], the process of interpretation can be seen as decoding and attaching meaning to the words and signs of a message. This decoding is filtered by the individual’s knowledge, experience, attitudes and context. Lindgren et al. [87] further describes interpretation as “a process that involves explaining, reframing, making sense of, or otherwise showing an understanding of… narratives”. Corey [86] describes this analysis of information within a transactional model of communication, where communication is seen as dynamic, and messages are interdependent. To build shared meaning, there must be some overlap in terms of culture, language, environment or experience. Since interpretation is essentially a subjective action, the nurse is dependent on establishing with the caller that the interpretations are correct in order to make a reliable assessment. Runius [14] refers to this as summarizing, an important feature of the last four phases of the telephone nursing dialogue process.

Forty-eight articles were found describing the *Motivating* phase of the telephone nursing dialogue process. The data corresponds to Runius’ [14] description of the content of this phase. However, the literature does not really align with the name of the phase. Whilst some articles describe the importance of motivating the triage decision to reach a mutual agreement about the plan of action [5, 31, 49], most of the articles do not mention the word “motivating”, but rather describe the advising of the caller. Greenberg [2]refers to this phase as the call output, encompassing intervention, support, collaboration and closing of the call. We would argue that nursing intervention could be seen as overarching all these concepts. The nursing intervention could consist of advice to see a specific caregiver (i.e. a referral), advice to perform self-care and advice to take certain medications, as well as safety-netting advice and informing, supporting and guiding the caller in such a way that the caller feels strengthened and secure in following the advice. We therefore suggest that this phase be renamed to better match the content of the phase as described in the literature.

An important remark about this phase is that the nurse does not make a triage decision about what the patient should do but rather makes a decision about what recommendation to give the caller depending on an assessment of the acuity, urgency and seriousness of the complaint. The plan of action is then developed in collaboration with the patient. However, the individual’s need for participation in the construction of an action plan might differ between individuals depending on the caller’s age, needs, health status, cognitive ability, cultural background and level of knowledge [88, 89].

**Fig. 3:**
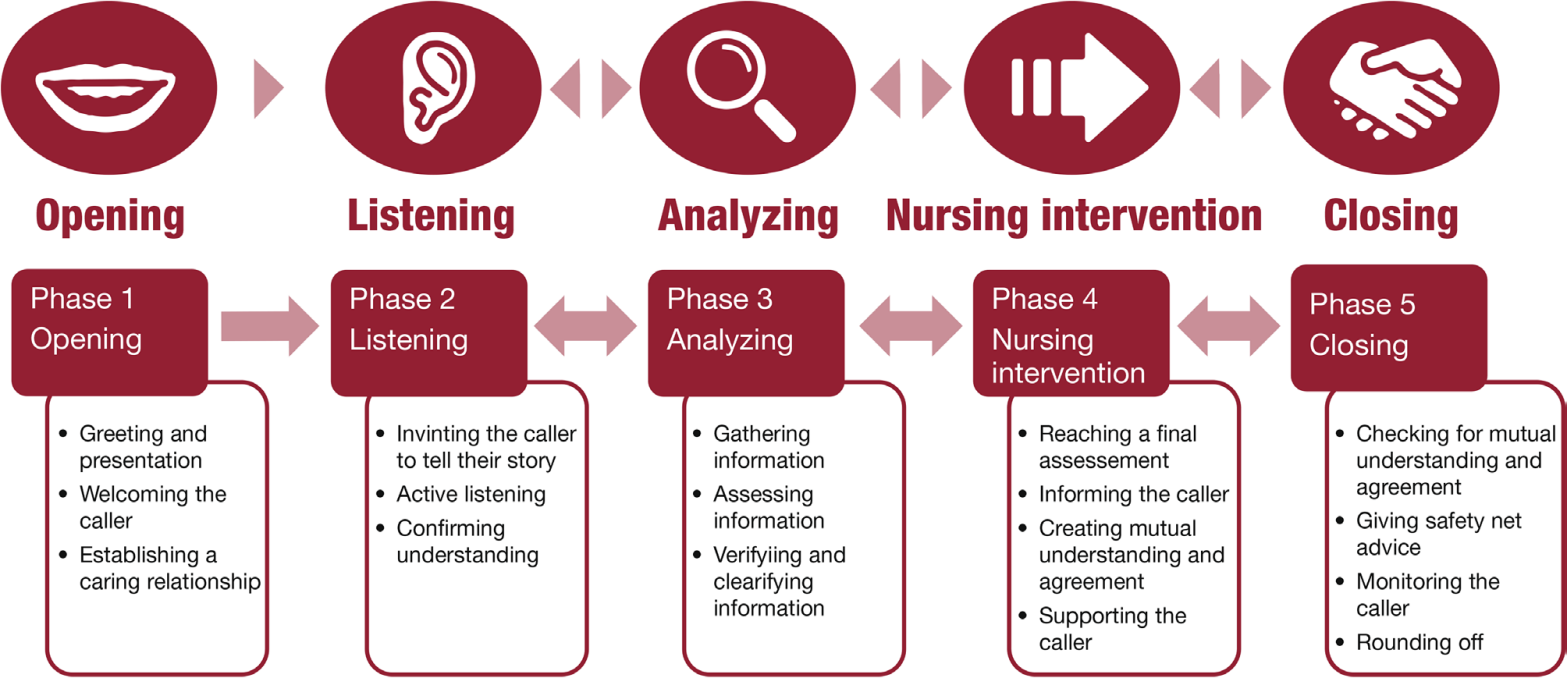
Model of the telephone nursing dialogue process

Finally, we found 35 articles describing the closing of the call. The data correspond to the methodological description [14]. Safety-netting and offering monitoring calls are important to make sure the caller is safe after ending the call, and the data as well as methodological literature emphasize the importance of continuously summarizing information to prevent communication failure [14]. The scientific literature mainly uses words such as verifying, repeating, clarifying, confirming understanding, and ensuring or checking for mutual agreement to describe this action, whilst the methodological literature refers to this as summarizing. Due to the inductive nature of the analysis, we have chosen to stay close to the data when naming the categories. But for pedagogical reasons, it could be wise to describe these actions under a common term, such as “confirming understanding” when teaching the model to elucidate the reciprocity and focus on the core concept. From the analysis of the literature, a suggestion for a revised model of the phases of the telephone nursing dialogue process was constructed (Fig. 3). This revised model follows the original model except for a change of the label “motivating” to “nursing intervention”, related to the findings discussed above, and with the addition of the subcategories found in our study. The revised model has many similarities to the TN process as described by Greenberg [2] in its dynamic nature and orientation towards meeting the caller’s needs, but it is more detailed and also iterative, where new information may necessitate transactional movement between phases during the call.

### Limitations

This study has limitations. Due to the heterogeneity of the studies included regarding design, aim and focus, a meta-analysis of the data was not possible. Da Silva et al. [90] advocate building a heterogeneous sample of literature describing the phenomenon of interest, since it is the pluralism of perspectives and methods that enables the development of a new conceptual comprehension. This is the reason why articles of both an empirical and a theoretical nature were included in this review. Despite the differences in perspectives, research methods and design, the findings were consistent throughout the studies, and similar constructs were sometimes described in both negative and positive terms (e.g. callers were satisfied when treated with respect/callers were dissatisfied when met with disrespect).

The literature search was extensive, broad search terms were used and the search generated large amounts of data. But even though the search was systematic and performed in four large databases, the risk of missing relevant literature is always present. According to Whittemore and Knafl [20], searching in computerized databases may yield only about 50% of eligible studies due to indexing problems and insufficient search terminology. A manual search of the reference lists of included articles was therefore performed to identify relevant literature that might otherwise have been missed. A decision was made to include earlier review studies which presented new synthesized findings. Accordingly, some of the articles included in these reviews were also used in the present review, which could be a limitation of this study.

The majority of included studies were from a Swedish context. This is not surprising given that TN is well researched in Sweden, but it might limit the transferability of the study findings due to cultural differences. Although nurses share a common professional history, internationally their educational preparation, regulation, and practice patterns are highly diverse and vary considerably in complexity and scope. There are differences in credentialing requirements that include professional licensure, use of titles, and accreditation of educational programs [91]. Another limitation of this review is that we have only been able to explore the scientific basis for the phases of the telephone nursing dialogue process as no studies of the process as a whole have yet been conducted.

A study protocol with a predetermined plan for the review methods was established prior to the onset of the review, but this protocol was not registered or published. No deviations from the protocol occurred.

## Conclusion

This study reveals a sound scientific basis for the phases of the telephone nursing dialogue process. The inductive categorization resulted in subcategories that align well with the methodological descriptions of the telephone nursing dialogue process. The exception is the phase “motivating,“ which would correspond more to the label “nursing intervention.“ The content of the data suggests an iterative process where new information can justify transactional movement within the process, as well as underlying structures such as the caring relationships that are continuously being shaped and reshaped depending on the nurse’s responses and actions. It is important to keep in mind that the entirety of the process has not yet been researched, indicating an important area for future research. It would also be of great significance to study whether using the telephone nursing dialogue process leads to improved nursing care and a decrease in medical errors and/or malpractice claims.

The telephone nursing dialogue process has the potential to enhance TN communication by promoting a structured exploration of a patient’s symptoms and context. The simplicity, holistic approach, and extensive clinical application of the telephone nursing dialogue process make it feasible in many different settings both nationally and internationally.

### Implications for practice

The study’s findings have several implications for clinical practice in telephone nursing. These implications emphasize the importance of establishing a positive opening of the call to create an alliance with the caller. Practicing active listening and verification techniques plays a vital role, enabling accurate information gathering while providing emotional support. Fostering collaborative decision-making enhances caller satisfaction and compliance, necessitating clear communication and adaptation to diverse situations and individual needs. Safety-netting advice ensures caller safety, with potential follow-up calls for ongoing monitoring and guidance. While computerized decision-support systems can aid, nurses’ clinical judgment remains essential. By integrating these principles into clinical practice, nurses can enhance the quality of telephone care and create efficient and compassionate telephone nursing. The continued development of communication, critical thinking, and clinical skills is essential for nurses to excel in providing quality care through telephone interactions.

## Data Availability

The data as well as detailed descriptions of the literature search, search outcome (including excluded articles) and the quality appraisal are available from the corresponding author upon request.
